# Cooperative contributions of Interferon regulatory factor 1 (IRF1) and IRF8 to interferon-γ-mediated cytotoxic effects on oligodendroglial progenitor cells

**DOI:** 10.1186/1742-2094-8-8

**Published:** 2011-01-24

**Authors:** Makoto Horiuchi, Aki Itoh, David Pleasure, Keiko Ozato, Takayuki Itoh

**Affiliations:** 1 The Department of Neurology, University of California Davis, School of Medicine, Sacramento, California, USA; 2 The Institute for Pediatric Regenerative Medicine, Shriners Hospitals for Children Northern California, Sacramento, California, USA; 3 The Laboratory of Molecular Growth Regulation, Program in Genomics of Differentiation, National Institute of Child Health and Human Development, National Institutes of Health, Bethesda, Maryland, USA

## Abstract

**Background:**

Administration of exogenous interferon-γ (IFNγ) aggravates the symptoms of multiple sclerosis (MS), whereas interferon-β (IFNβ) is used for treatment of MS patients. We previously demonstrated that IFNγ induces apoptosis of oligodendroglial progenitor cells (OPCs), suggesting that IFNγ is more toxic to OPCs than IFNβ. Thus we hypothesized that a difference in expression profiles between IFNγ-inducible and IFNβ-inducible genes in OPCs would predict the genes responsible for IFNγ-mediated cytotoxic effects on OPCs. We have tested this hypothesis particularly focusing on the interferon regulatory factors (IRFs) well-known transcription factors up-regulated by IFNs.

**Methods:**

Highly pure primary rat OPC cultures were treated with IFNγ and IFNβ. Cell death and proliferation were assessed by MTT reduction, caspse-3-like proteinase activity, Annexin-V binding, mitochondrial membrane potential, and BrdU-incorporation. Induction of all nine IRFs was comprehensively compared by quantitative PCR between IFNγ-treated and IFNβ-treated OPCs. IRFs more strongly induced by IFNγ than by IFNβ were selected, and tested for their ability to induce OPC apoptosis by overexpression and by inhibition by dominant-negative proteins or small interference RNA either in the presence or absence of IFNγ.

**Results:**

Unlike IFNγ, IFNβ did not induce apoptosis of OPCs. Among nine IRFs, IRF1 and IRF8 were preferentially up-regulated by IFNγ. In contrast, IRF7 was more robustly induced by IFNβ than by IFNγ. Overexpressed IRF1 elicited apoptosis of OPCs, and a dominant negative IRF1 protein partially protected OPCs from IFNγ-induced apoptosis, indicating a substantial contribution of IRF1 to IFNγ-induced OPC apoptosis. On the other hand, overexpression of IRF8 itself had only marginal proapoptotic effects. However, overexpressed IRF8 enhanced the IFNγ-induced cytotoxicity and the proapoptotic effect of overexpressed IRF1, and down-regulation of IRF8 by siRNA partially but significantly reduced preapoptotic cells after treatment with IFNγ, suggesting that IRF8 cooperatively enhances IFNγ-induced OPC apoptosis.

**Conclusions:**

This study has identified that IRF1 and IRF8 mediate IFNγ-signaling leading to OPC apoptosis. Therapies targeting at these transcription factors and their target genes could reduce IFNγ-induced OPC loss and thereby enhance remyelination in MS patients.

## Background

Persistent demyelination often follows recurrent inflammation in multiple sclerosis (MS), even though oligodendroglial progenitor cells (OPCs) are present in the adult CNS as a potential source of oligodendrocytes for remyelination after loss of myelin [1-3]. As a pathological mechanism underlying this remyelination failure, accumulating evidence indicates that interferon-γ (IFNγ), the only type II IFN secreted into the lesions by infiltrating T helper 1 (T_H_1) cells and natural killer cells, induces cytotoxic effects on OPCs, and inhibits their differentiation, leading to failure in *de novo *myelination by OPCs [4-9]. We also demonstrated in our previous study that actively proliferating OPCs are far more susceptible to cytotoxic effects of IFNγ than are post-mitotic mature myelinating oligodendrocytes [10]. In contrast to IFNγ, interferon-β (IFNβ), a type I IFN, is used successfully to reduce relapse rates in relapsing remitting MS [11]. However, though IFNβ has minimal adverse effects on proliferation, migration and differentiation of oligodendrocytes *in vitro *[12,13], it does inhibit remyelination after cuprizone-induced demyelination *in vivo *[14]. Given the extensive overlap in type I and type II IFN signaling pathways, our goal in the present study was to determine what molecular mechanisms are responsible for the much greater OPC toxicity of IFNγ than IFNβ.

The janus kinase (JAK)/signal transducer and activator of transcription (STAT) pathway has been well-studied as a principal intracellular signaling pathway activated by IFNs (Reviewed in [15]). Binding of IFNγ to the type II IFN receptor results in the rapid autophosphorylation and activation of the receptor-associated JAK1 and JAK2, which in turn activates cytoplasmic STAT1 by phosphorylation at Tyr 701. Activated STAT1 proteins form homodimers, which translocate into the nucleus, and initiate transcription by binding to a specific motif, known as the IFNγ-activated site (GAS), in the promoters of various IFN-stimulated genes (ISGs). IFNβ utilizes another receptor, the type I IFN receptor, associated with JAK1 and tyrosine kinase 2 (TYK2), and regulates formation of the heterotrimeric transcription complex, interferon-stimulated gene factor 3 (ISGF3), composed of the activated forms of STAT1 and STAT2, and IRF9/ISGF3γ. ISGF3 recognizes the IFN-stimulated response element (ISRE) which is distinct from the GAS, and activates transcription of another set of ISGs. Although there is a substantial overlap between IFNβ-inducible and IFNγ-inducible ISGs stemming from their common dependence on activation of STAT1 [16], we hypothesized that IFNγ-mediated cytotoxic effects on OPCs are attributable to ISGs which are differently induced in OPCs by IFNγ than by IFNβ.

As an example of the ISGs which are differently induced between IFNγ and IFNβ, we previously examined IFN-mediated transcriptional induction of major histocompatibility complex class II (MHC-II) molecules in the oligodendroglial lineage [17]. Surface expression of MHC-II becomes detectable in OPCs after treatment with IFNγ, whereas IFNβ fails to induce expression of MHC-II. Our results indicated that the distinct difference in transcriptional activation of interferon regulatory factor 1 (IRF1) between IFNγ and IFNβ is attributed to the difference in subsequent MHC-II expression. Thus, IRF1 is also a promising example of the ISGs responsible for IFNγ-mediated cytotoxic effects in OPCs. In agreement with this idea, involvement of IRF1 in IFNγ-induced OPC apoptosis has recently been reported [18].

IRF1 was originally isolated as a transcriptional activator of the IFNβ gene in response to viral infection [19,20]. IRF1 and eight other subsequently identified factors share a highly-conserved amino-terminal DNA binding domain (DBD) with five conserved tryptophan repeats, and thereby constitute a family of transcription factors, termed the IRF family (Reviewed in [21-24]). The DBD forms a helix-turn-helix domain and recognizes similar, if not identical, DNA motifs containing the consensus IRF recognition sequence, 5'-AANNGAAA-3' [25]. IRF2, which shares the highly homologous DBD with IRF1, is considered a transcriptional repressor for IRF1-mediated transcription by competing for the same *cis *elements [19,26,27]. In addition, most of the members, except IRF1 and IRF2, have an IRF association domain (IAD) at the C-terminal region, through which they interact with other members or other transcription factors. Despite the possible functional overlap and interplay among members of the IRF protein family, however, there have been only a few studies on the IRF family members in the oligodendroglial lineage [28], particularly with respect to their roles in IFNγ-mediated and IFNβ-mediated signaling [17]. In this study, using primary cultures of highly pure OPCs from rats, we performed a comprehensive analysis of all members of the IRF family in OPCs in response to IFNγ and IFNβ, and examined the synergistic roles for IRF1 and IRF8 (also known as interferon consensus sequence binding protein (ICSBP)), in IFNγ-induced OPC apoptosis.

## Methods

### Reagents and chemicals

All reagents and culture media used in this study were purchased from SIGMA (St. Louis, MO, USA) and Invitrogen (Carlsbad, CA, USA), respectively, except for the following products: Human recombinant fibroblast growth factor 2 and platelet-derived growth factor A homodimer were from R&D systems (Minneapolis, MN, USA); rabbit anti-IRF1 and anti-IRF2 antibodies were from Santa Cruz Biotechnology (Santa Cruz, CA, USA), and mouse anti-glyceraldehyde-3-phosphate dehydrogenase (GAPDH) antibodies were from Chemicon (Temecula, CA, USA). Rabbit anti-IRF8 antibody was produced by Ozato's laboratory. Small interfering RNA (siRNA) for IRF2 (siRNA ID: s220597), IRF8 (siRNA ID: s146232), and Negative control siRNA were from Ambion (Austin, TX, USA).

### Mixed glial culture

Primary mixed glial cultures from rats were prepared as reported previously [29]. Briefly, whole brains were dissected from 0 to 2-day-old Lewis rats, and submerged in ice-cold Leibovitz's L-15 medium. Under a dissecting microscope, olfactory bulbs, cerebral cortices and hindbrains were removed. After cleaning off meninges and vessels including choroidal plexus, the remaining brain tissues were cut into small chunks with a 21-gauge needle, and digested by 0.0625% (w/v) trypsin in Ca^2+ ^and Mg^2+^-free Hank's Balanced Salt Solution (HBSS) for 20 min. Dissociated cells were obtained by passing the softened chunks through a 1 ml pipette tip several times, and collected by centrifugation at 365 xg for 5 min. The cells were resuspended in minimum essential medium alpha containing 5% (v/v) fetal bovine serum and 5% (v/v) calf serum, and plated onto a 10 cm culture dish. One day after plating, attached cells (designated as passage 0) were washed with HBSS to remove serum, and thereafter maintained in the medium (GM), a 3:7 mixture (v:v) of B104 neuroblastoma-conditioned medium and the N1 medium (high glucose Dulbecco's modified Eagle's medium supplemented with 6 mM _L_-glutamine, 10 ng/ml biotin, 5 μg/ml insulin, 50 μg/ml apo-transferrin, 30 nM sodium selenite, 20 nM progesterone and 100 μM putrescine as final concentrations). Cultures were fed with fresh GM medium every other day for approximately 5 days, at which time the proliferating glial cells were almost confluent.

### Immunopanning for purification of A2B5^+ ^rat OPCs

The mixed glial cultures were washed with Ca^2+ ^and Mg^2+^-free HBSS, suspended in the N1 medium containing 0.1% (w/v) BSA, and plated and incubated on negative immunopanning plates coated with RAN-2 antibody for 30 min at 37°C to exclude RAN-2-positive cells [29]. Following two rounds of this negative selection, nonadherent cells were transferred to the A2B5 positive panning plates. After the serial immunopanning, purified cultures contained more than 95% of OPCs which were A2B5-positive, O4-negative, and glial fibrillary acidic protein-negative.

### Immunocytochemistry

Cells cultured on poly-_D_-lysine-coated coverslips were incubated with A2B5 hybridoma supernatants (undiluted) at room temperature for 30 min. After washing with phosphate-buffered saline (PBS), cells were fixed with 4% paraformaldehyde at room temperature for 15 min, and then permeabilized with 100% methanol at -20°C for 15 min. For IRF8 staining, cells were incubated with anti-IRF8 antibody diluted at 1:50 in PBS containing 5% normal goat serum and 0.03% Triton-X 100 at room temperature for 2 h, after permeabilization by 100% methanol. After incubation with fluorophore-conjugated secondary antibodies (1:50, v:v) in PBS at room temperature for 30 min, nuclei were counterstained with 4,6-diamidio-2-phenylindole (0.5 μg/ml) for 10 min, and then the coverslips were mounted on slide glasses with VectorShield (Vector laboratory, Burlingame, CA, USA).

### Immunoblots

Protein lysates were prepared in the lysis buffer as described previously [10]. Twenty μg of protein from each sample were size-fractioned by SDS-polyacrylamide gel electrophoresis, transferred onto a nitrocellulose membrane (Schleicher & Schnell, Keene, NH, USA) and probed with primary antibodies for IRF1 (1:400, v:v) and IRF8 (1:5000, v:v) for 1 h. Full range recombinant Rainbow Molecular Weight Markers (Amersham Biosciences, Piscataway, NJ, USA) were used as a reference for molecular sizes. Immunoreactive signals were detected by enhanced chemiluminescence according to the manufacture's protocol (Amersham Biosciences). Equal protein loading was confirmed by subsequent probing with the mouse monoclonal antibody against GAPDH in each experiment.

### Caspase activity assay

Cells were homogenized in lysis buffer (100 mM HEPES; 10% (w/v) sucrose; 0.1% (w/v) 3-[(3-cholamidopropyl) dimethylammonio]-1-propanesulfonate; 10 mM dithiothreitol; 1 mM EDTA; 1 mM phenylmethylsulfonyl fluoride, 2 μg/ml aprotinin; 1 μg/ml pepstatin; 5 μg/ml leupeptin) [30]. The protein lysates were stored at -80°C until use as a 1:1 (v:v) mixture with glycerol. Caspase activity was measured by a fluorometric method; protein samples (10 μg) were incubated with the fluorogenic substrate, acetyl-Asp-Glu-Val-Asp-α-(4-methylcoumaryl-7-amide) (12.5 μM) (Ac-DEVD-AMC, Peptides international, Luoisville, KY, USA) in 250 μl of the lysis buffer, and cleavage of Ac-DEVD-AMC was monitored by a multiplate spectrofluoromater, Gemini EM (Molecular devices, Sunnyvale, CA, USA) for 60 min at 25°C. The DEVD-cleavage activity was expressed as delta RFU (relative fluorescence unit)/μg protein/h.

### 5-bromo-2'-deoxyuridine (BrdU)-incorporation assay

OPCs cultured in 60 mm dishes were exposed to a 4 h BrdU pulse (10 μM) just prior to harvesting. The trypsinized cells were collected in GM and resuspended in 1.5 ml PBS. After fixation by 70% (v/v) ethanol at -20°C for overnight, 5 × 10^4 ^cells were washed with 1 ml of the washing buffer (0.1% (w/v) BSA in PBS), and denatured by resuspension in 2N HCl at room temperature for 20 min. After resuspending once more in washing buffer, the cells were incubated in 0.1 M sodium borate at room temperature for 2 min to neutralize any residual acid. Cells that had incorporated BrdU following incubation were identified by incubation with a fluorescein isothiocyanate (FITC)-conjugated mouse anti-BrdU monoclonal antibody at room temperature for 20 min in dilution buffer (0.1% (w/v) BSA, 0.5% (v/v) Tween-20 in PBS) followed by another resuspension in washing buffer. The labeled cells were detected in the green (FL1) channel of a flow cytometer, CyAn-ADP (Dako cytomation, Carpinteria, CA). FITC-conjugated mouse monoclonal IgG1 was used as isotype control.

### MTT assay

Cell viability was estimated by the enzymatic conversion of 3-(4,5-dimethylthiazol-2-yl)-2,5-diphenyltetrazolium bromide (MTT) to formazan crystals in live cells. Formazan was dissolved in dimethyl sulfoxide at 90 min after addition of MTT (0.5 mg/ml) to the culture medium, and quantified by a spectrophotometer or a microplate reader at 560 nm.

### Annexin-V and propidium iodide binding assay

The OPC cultures were maintained in 60 mm dishes and subjected to various experimental treatments. At 0, 24, and 48 h after these treatments, the culture medium containing detached dead cells was collected, and the attached cells were washed once with 2 ml of Ca^2+ ^and Mg^2+^-free HBSS. The attached cells were removed from the plate by exposure to 0.5 ml of 0.05% trypsin at 37°C for 2 min, suspended in 2 ml of GM with 625 μg/ml trypsin inhibitor, and collected into a 15 ml tube together with the saved medium and the Ca^2+ ^and Mg^2+^-free HBSS used for wash. After centrifuge at 520 xg for 10 min, the pellet was resuspended into 0.4 ml of binding buffer (0.1 M HEPES, pH 7.4; 140 mM NaCl; 2.5 mM CaCl_2_; 0.45% (w/v) _D_-glucose). Five μl of FITC-conjugated annexin-V solution and propidium iodide (PI; 8 μg/ml at final concentration) were added into 0.1 ml of the cell suspension. After incubation at room temperature for 15 min in the dark, 0.3 ml of the binding buffer was added to the cell suspension. To determine the absolute number of cells in each preparation, Flow-Count™ fluorospheres were added at a concentration of 19 beads/μl just before flow cytometry by CyAn-ADP (DakoCytomation, Carpinteria, CA, USA). Fluorescence of annexin-V-FITC and PI were detected in FL-1 and FL-4 channels, respectively. Gatings and data acquisition and analysis were carried out using Summit software (DakoCytomation) as described previously [10].

### Cell death and loss of mitochondrial membrane potential assay

Rat OPCs cultured in 24-well plates were treated with the GM or the GM supplemented with IFNγ for 12, 18, and 24 h. Prior to collection, cells were incubated with tetramethylrhodamine ethyl ester (TMRE, 0.1 μM) at 37°C for 30 min. Then, culture medium containing dead cells was collected, and cells were washed once with 0.5 ml of the Ca^2+ ^and Mg^2+^-free HBSS. Attached cells were removed with 150 μl of 0.05% trypsin for 1 min, suspended in 1 ml of the GM, and collected into a 15-ml tube together with the saved medium and the Ca^2+ ^and Mg^2+^-free HBSS used for washing. After centrifugation with 1500 rpm for 5 min, the supernatant was aspirated and the pellet was kept on ice. Pellets were resuspended with 0.5 ml PBS containing 5 μM DAPI and 0.1% BSA immediately prior to analysis by flow cytometry employing a Cyan-ADP Flow Cytometer (DakoCytomation). Live and dead cell populations were gated as described previously [10], and TMRE and DAPI were detected in the FL-2 and the FL-6, respectively.

### Real-time PCR

Real-time PCR analyses were performed by MX3005P (Stratagene, La Jolla, CA, USA) using TaqMan^® ^Assay-on-Demand™ assay kits (assay nos.: Rn00561424_m1, Mm00515204_m1, Rn01764369_m1, Rn01435145_m1, Rn01500522_m1, Rn01762216_g1 and Rn01751474_m1 for detection of IRF1, IRF2, IRF3, IRF4, IRF5, IRF8 and interferon gamma induced GTPase (IGTP) cDNA, respectively) [17]. For detection of IRF6, IRF7, and IRF9 cDNA, each set of primers and a probe was obtained from Applied Biosystems as a Custom TaqMan^® ^Gene Expression Assay, because the kits for these cDNA were not available at the time of these experiments. For standardization, GAPDH cDNA levels were quantified with TaqMan Rodent GAPDH Control Reagents according to the manufacturer's instructions, and the absolute cDNA amounts were expressed as ratios to GAPDH cDNA. We present representative data from at least two independent analyses for each mRNA.

### Plasmid construction

For the forced expression vectors, the open reading frame of rat IRF1 or IRF8 was inserted in the pcDNA3.1 mammalian expression vector followed by the internal ribosome entry site (IRES) and humanized Renilla reniformis green fluorescent protein (hrGFP, Stratagene) in order to facilitate identification of transfected cells by flow cytometry or by fluorescence microscopy. The expression vector for the dominant-negative form of IRF1 (IRF1DN-hrGFP) and was constructed by inserting the coding sequence of truncated rat IRF1 (amino acids 1 to 144) into the pcDNA3.1 mammalian expression vector. The truncated IRF1 coding sequence was fused to hrGFP in frame with a spacer sequence, Pro-Gly-Gly-Gly-Gly-Pro (P4GP) hinge, in order to facilitate identification of transfected cells and to evaluate the intracellular localization and stability of the dominant negative protein. For forced double expression of IRF1 and IRF8, the expression construct for IRF8 lacking hrGFP reporter (PCMV-IE-IRF8-pA) was prepared by inserting the open reading frame of rat IRF8 into pcDNA3.1.

### Transfection by electroporation

Trypsinized OPCs (2 × 10^6^) were resuspended in 100 μl of N1 medium with 10 μg plasmid DNA or 2 μM siRNA, and put into a 2 mm cuvette. A square pulse with 110 mV for 25 msec was applied to the mixture of cells and plasmid DNA with BioRad GenePulser Xcell (BioRad, Hercules, CA, USA). Cells were resuspended into GM, plated on 24 well plates, and subjected to further experimental procedures.

### Statistical analysis

Data are presented as mean ± SD unless otherwise noted. Statistical significance was determined by two-tailed ANOVA followed by Student-Newman-Keuls post hoc test.

## Results

### IFNβ is far less cytotoxic to OPCs than IFNγ

**Figure 1:**
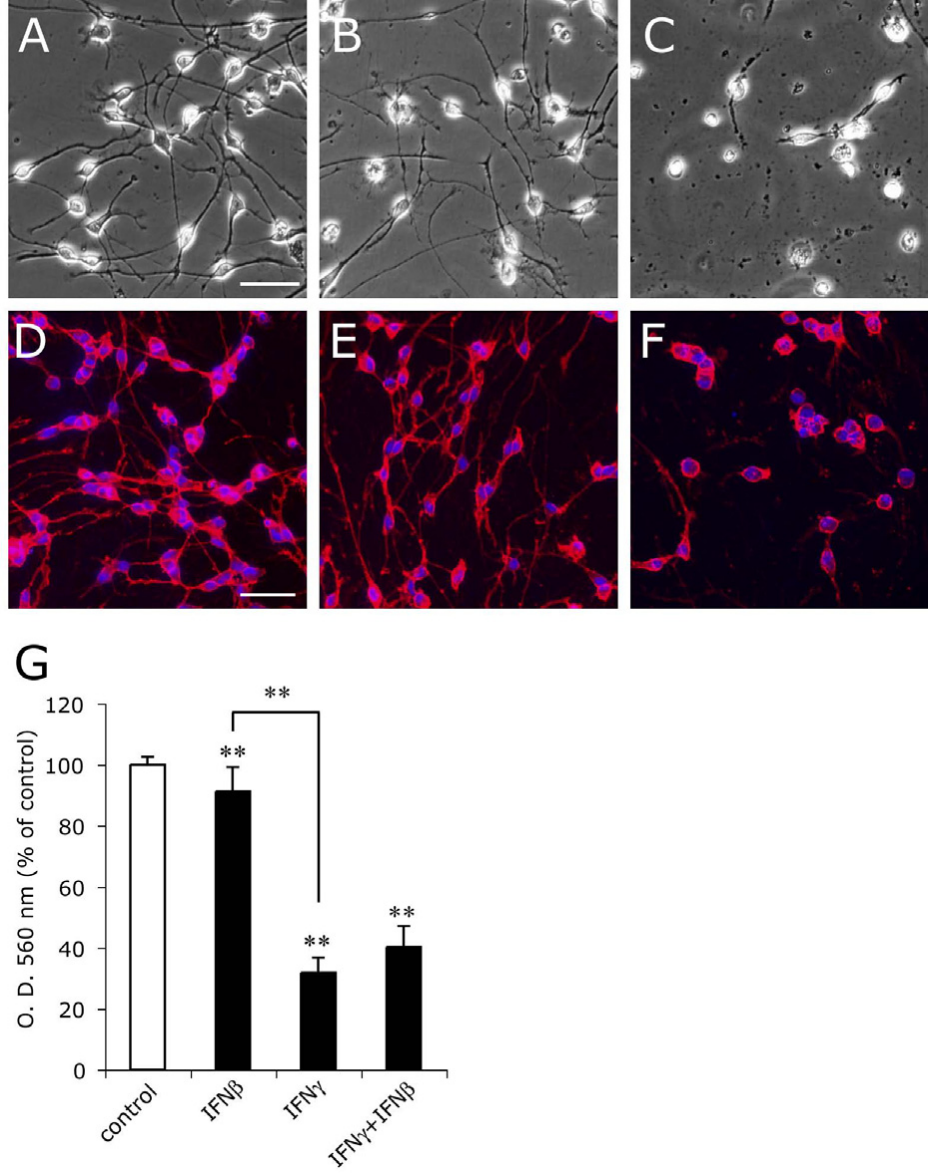
**IFNβ is far less toxic to OPCs than IFNγ**. Phase-contrast images (**A**-**C**) and immunocytochemistry for A2B5 (**D**-**F**) of rat OPCs treated with GM alone (**A**, **D**), GM plus IFNβ (1 kU/ml) **(B**, **E**) or GM plus IFNγ (100 ng/ml) (**C**, **F**) for 48 h. **G**, Viability of OPCs cultured with GM (control), GM supplemented with IFNβ (IFNβ), IFNγ (IFNγ), or both (IFNγ + IFNβ) was measured by MTT assay at 48 h after treatment. ** Indicates p < 0.01 compared with control. ** Indicates p < 0.01 compared with control or in comparison between the two groups indicated.

IFNγ significantly reduced the viability of purified A2B5-positive OPCs to 32 ± 5% of the controls at 48 h as reported in our prior study [10]. In contrast, IFNβ decreased the viability only to 91 ± 8% of the controls at 1 kU/ml, a concentration sufficient to exert maximum biological effects in various cell types [13,31,32] (Figure. 1G). IFNβ failed to protect OPCs from IFNγ-induced cytotoxicity when IFNβ and IFNγ were added simultaneously (Figure. 1G). IFNβ did not alter surface expression of A2B5 or the typical OPC morphology (Figure. 1A-F).

**Figure 2:**
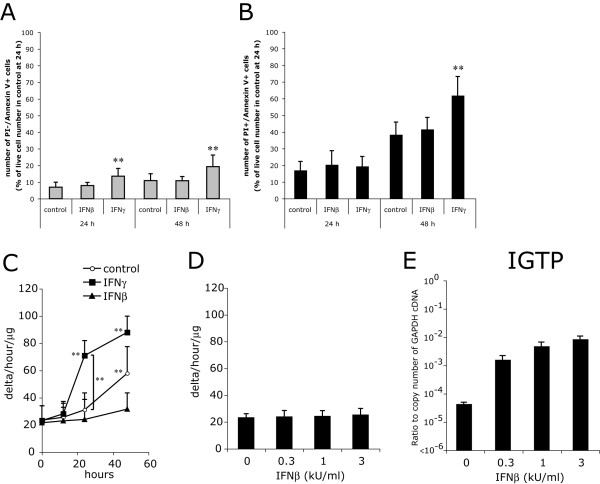
**Unlike IFNγ, IFNβ does not induce OPC apoptosis**. **A**-**B**, Numbers of Annexin-V^+^/PI^- ^(preapoptotic) cells and Annexin-V^+^/PI^+ ^(dead) OPCs after treatment with IFNβ or IFNγ for 24h and 48 h. Annexin-V^-^/PI^- ^(live), preapoptotic, and dead cells were counted by flow cytometry, and shown as percentages of averaged live cell numbers in control at 24 h. **C**, Caspase-3-like protease activity was measured with the fluorogenic substrate, Ac-DEVD-MCA, in the protein lysates of OPCs treated with GM alone (control, open circles), IFNβ (closed triangles) or IFNγ (closed circles) for 12, 24, and 48 h. ** indicates p < 0.01 compared with control or in comparison between the two groups indicated. **D**-**E**, Dose-dependent effects of IFNβ on induction of caspase-3-like proteinase activity and IGTP mRNA in OPCs. OPCs were treated with IFNβ at 0, 0.3, 1, and 3 kU/ml for 24 h, and activity of caspase-3-like proteinase (**D**) and IGTP mRNA (**E**) were quantified.

Cytotoxicity of IFNγ to OPCs consists of increase in apoptosis and delay in G1/S transition of the cell cycle [10]. Double staining with Annexin-V-FITC and PI revealed that IFNβ did not increase numbers of preapoptotic and dead cells compared to the control OPC cultures, whereas preapoptotic cells became detectable from 24 hr, and dead cells were significantly increased at 48 h in the IFNγ-treated OPC cultures (Figure. 2A, B). Caspase-3-like protease activity was significantly induced by IFNγ as early as 24 h, but not by IFNβ even at 48 h (Figure. 2C). These results indicated that, unlike IFNγ, IFNβ did not enhance OPC apoptosis.

This far less proapoptotic effect of IFNβ on OPCs was not a consequence of less equivalent biological activity of IFNβ at 1 kU/ml compared to IFNγ at 100 ng/ml. First, a higher concentration of IFNβ, 3 kU/ml, also failed to induce caspase-3-like protease activity in OPCs (Figure. 2D). Second, as far as determined by transcriptional induction of IFNγ induced GTPase (IGTP), IFNβ at 0.3 kU/ml or higher was sufficient to induce maximal levels of IGTP mRNA (Figure. 2E). Third, based on the standard anti-viral assay to measure the biological activities of IFNs [33], 100 ng/ml IFNγ corresponds to approximately 0.1 to 1 kU/ml, which is almost comparable to the biological unit of IFNβ used in this study. We therefore compared the effects of IFNγ and IFNβ at 100 ng/ml and 1 kU/ml, respectively, in further experiments.

**Figure 3:**
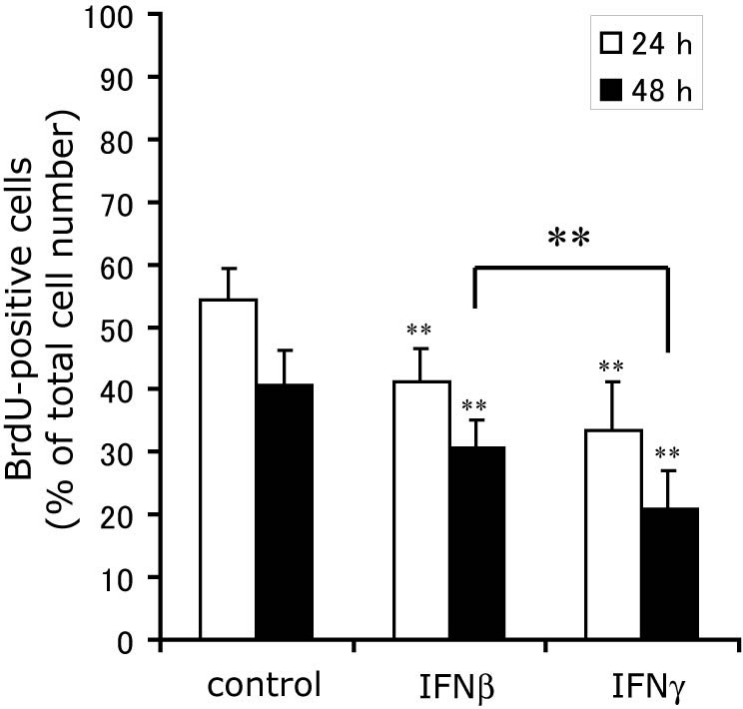
**IFNβ inhibits progression of the cell cycle, though to a lesser extent than IFNγ**. BrdU-incorporation of OPCs incubated with IFNβ (1 kU/ml, IFNβ) or IFNγ (100 ng/ml, IFNγ) for 24 (open bars) and 48 h (closed bars). OPCs were exposed to a 4 h-BrdU pulse immediately before fixation. ** Indicates p < 0.01 compared with control or in comparison between the two groups indicated.

IFNγ has been shown to inhibit cell cycle progression in OPCs as well [10]. Cells were exposed to a 4 h BrdU pulse immediately prior to fixation at 24 and 48 h after treatments with IFNβ or IFNγ. The results confirmed that both IFNγ and IFNβ significantly slowed progression of the cell cycle (p < 0.01) at 24 and 48 h. Percentages of BrdU-positive cells were significantly lower in the OPCs treated with IFNγ than those treated with IFNβ at 48 h, indicating that IFNβ did inhibit cell cycle progression in OPCs, but to a lesser extent than IFNγ (Figure. 3).

### Depolarization of the mitochondrial membrane potential precedes IFNγ-induced OPC apoptosis

**Figure 4:**
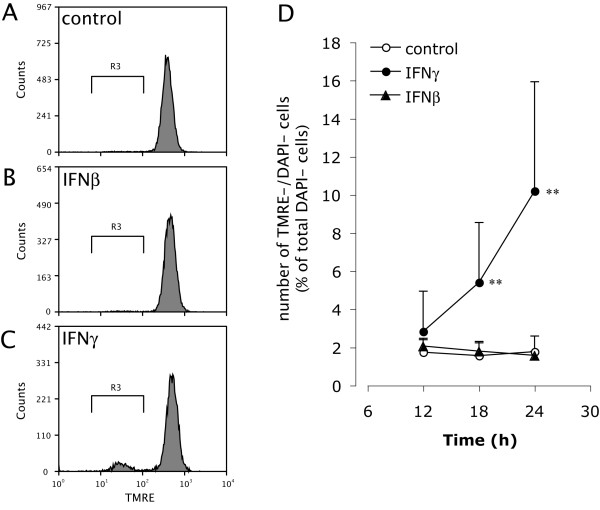
**IFNγ, but not IFNβ, depolarizes OPC mitochondria prior to loss of plasma membrane integrity**. Representative histograms of TMRE signals in DAPI-negative OPCs at 24 h after treatment with GM alone (control, **A**), IFNβ (**B**) or IFNγ (**C**). More TMRE^-^/DAPI^- ^OPCs were detected in the gated area (R3) in the cultures treated with IFNγ for 24 h compared to control and IFNβ-treated cultures. **D**, TMRE^-^/DAPI^- ^OPCs became detectable between 12 and 18 h after treatment with IFNγ (closed circle). In contrast, GM alone (control, open circles) or IFNβ (closed triangles) did not increase this population until 24 h after treatment. ** Indicates p < 0.01 compared with control.

Depolarization of the mitochondrial inner membrane is one of the earliest hallmarks of apoptosis in many cell types [34,35]. We identified preapoptotic OPCs with depolarized mitochondria in the IFNγ- and IFNβ-treated OPC cultures by live cell staining with both TMRE and DAPI followed by flow cytometry. The number of preapoptotic cells with depolarized mitochondria but retaining an intact plasma membrane (TMRE^-^/DAPI^- ^cells) was significantly increased in OPC cultures treated with IFNγ as early as 18 h after treatment, confirming that mitochondrial depolarization preceded IFNγ-induced OPC apoptosis. In good agreement with the results of the viability and caspase activity assays, however, preapoptotic OPCs did not increase in the cultures treated with IFNβ (Figure. 4).

### IRF1 and IRF8 are preferentially up-regulated in OPCs treated with IFNγ compared to those treated with IFNβ

**Figure 5:**
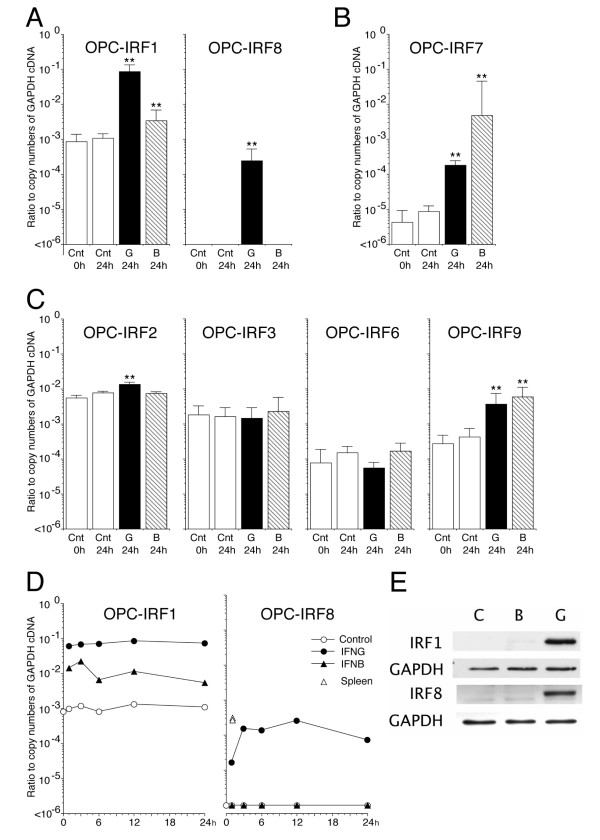
**IRF1 and IRF8 are preferentially up-regulated in OPCs treated with IFNγ**. **A**-**C**, Quantitative analysis of induction of IRF1, IRF2, IRF3, IRF6, IRF7, IRF8, and IRF9 mRNA in OPCs before (Cnt 0 h) and at 24 h after incubation with IFNγ (100 ng/ml, G 24 h), IFNβ (1 kU/ml, B 24 h), or medium alone (Cnt 24 h). Each data point was from at least 3 independent experiments. Note that the data are plotted as ratios to copy numbers of GAPDH cDNA on a logarithmic scale. ** Indicates p < 0.01 compared with control at 24 h (Cnt 24 h). **D**, IRF1 and IRF8 mRNA in OPCs were quantified by real-time PCR at 1, 3, 6, 12, and 24 h after addition of GM alone (control, open circle), IFNγ (100 ng/ml, closed circle) or IFNβ (1 kU/ml, closed triangle). For IRF8, basal IRF8 mRNA levels in the two RNA samples of spleen are shown as positive control (open triangles). At time 0, data from controls are only shown. **E**, Induction of IRF1 and IRF8 proteins in OPCs was examined at 24 h after treatment with IFNγ and IFNβ by immunoblotting.

IFNγ induces OPC apoptosis, while IFNβ does not. We hypothesized that, although IFNβ and IFNγ transcriptionally up-regulate substantially overlapping ISGs [16], there must be some critical difference between IFNγ-inducible and IFNβ-inducible gene sets that is responsible for IFNγ-induced apoptosis of OPCs. Among hundreds of ISGs, some members of the IRF protein family are immediate transcriptional targets of interferon-mediated JAK/STAT signaling, and subsequently control induction of downstream ISGs as transcription regulators [21-24]. Indeed, we previously demonstrated that IRF1 and IRF9 transcriptional kinetics differ between IFNγ-treated and IFNβ-treated OPCs [17]. IFNγ elicited a more than 70-fold sustained elevation of IRF1 mRNA from the basal levels in OPCs. In contrast, IFNβ-mediated up-regulation of IRF1 mRNA was transient even in the continuous presence of IFNβ, falling to less than one tenth of the sustained levels induced by IFNγ at 24 h. We extended this analysis to other members of the IRF protein family to obtain a comprehensive view of differential transcriptional regulation of all known IRFs in response to IFNγ and IFNβ, because at least some members are able to heterodimerize [36-38]. The quantitative PCR results demonstrated that members of the IRF protein family in OPCs could be classified into three groups in terms of their distinctive patterns of transcriptional induction by IFNγ and IFNβ; 1) IRF1 and IRF8 were preferentially up-regulated by IFNγ compared with IFNβ (Figure. 5A), 2) IRF7 was preferentially up-regulated by IFNβ compared with IFNγ (Figure. 5B), and 3) IRF2 to IRF6 and IRF9 were similarly regulated or not regulated by IFNγ and IFNβ, with the basal levels of the transcripts being IRF2 > IRF3 > IRF9 > IRF6 > IRF5 (Figure. 5C, The results of IRF5 are not shown.). IRF4 mRNA was below the detection limit in OPCs even in the presence of IFNs. We therefore focused on roles for IRF1 and IRF8 in IFNγ-induced apoptosis of OPCs in this study, because IRF1 mRNA and IRF8 mRNA were up-regulated within 1 hr after addition of IFNγ, and remained at more than 10-fold higher levels than those induced by IFNβ until at least 24 h (Figure. 5D). Immunoblotting for IRF1 and IRF8 proteins also confirmed selective up-regulation of these proteins in the IFNγ-treated OPC cultures (Figure. 5E).

### IRF1 mediates IFNγ-induced OPC apoptosis

**Figure 6:**
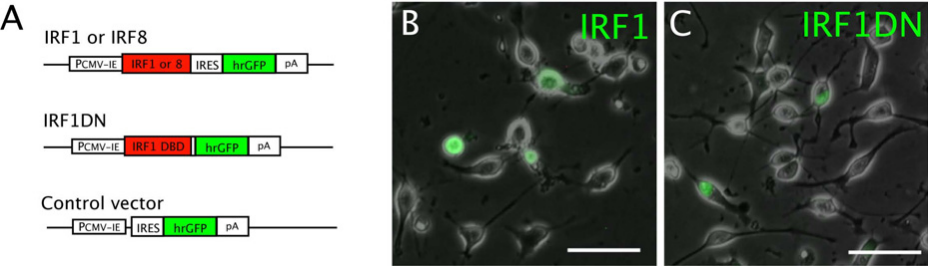
**Expression constructs used in the study**. **A**, PCMV-IE, immediate-early cytomegalovirus promoter; IRF1 DBD, IRF1 DNA binding domain; IRES, internal ribosome entry site; hrGFP, humanized Renilla GFP; pA, poly adenylation signal sequence. **B**, Fluorescence signals of hrGFP in the transfected cells became detectable by microscopy as early as 6 h after transfection. **C**, Fluorescent imaging demonstrates that the IRF1DN-hrGFP fusion protein was localized in the nuclei of transfected OPCs.

**Figure 7:**
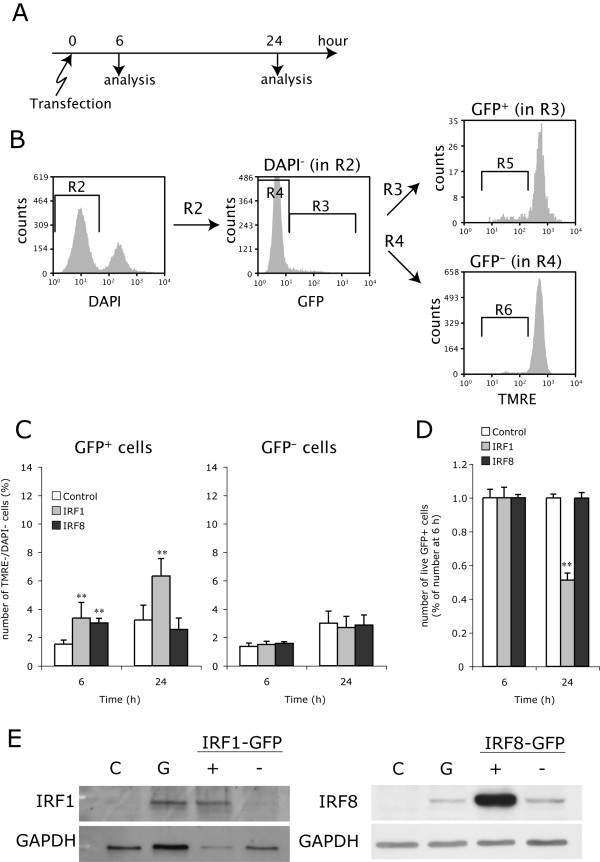
**Overexpressed IRF1 induces OPC apoptosis, whereas overexpressed IRF8 does not**. **A**, OPCs were transfected with the control vector (PCMV-IE-IRES-hrGFP-pA), the expression construct of rat IRF1 (PCMV-IE-IRF1-IRES-hrGFP-pA), or the expression construct of rat IRF8 (PCMV-IE-IRF8-IRES-hrGFP-pA) by electroporation. These OPCs were incubated with TMRE and DAPI at 6 and 24 h after transfection and analyzed by flow cytometry. **B**, Representative gating scheme of flow cytometric analysis of preapoptotic (TMRE^-^/DAPI^-^) OPCs in the transfected (hrGFP^+^) and untransfected (hrGFP^-^) populations at 24 h after transfection with the IRF1 expression construct. Live cells negative for DAPI in the R2 gate were separated into hrGFP ^+ ^(R3) and hrGFP ^- ^(R4) cells. Preapoptotic OPCs were then counted in the R5 and R6 gated areas in hrGFP ^+ ^(R3) and hrGFP ^- ^(R4) populations, respectively. **C**, Number of preapoptotic (TMRE^-^/DAPI^-^) OPCs in transfected (hrGFP^+^) and untransfected (hrGFP^-^) populations at 6 and 24 h after transfection with the control vector, IRF1 expression construct or IRF8 expression construct. **D**, Reduction in live transfected cells (DAPI^-^/hrGFP^+^) at 24 h in the cultures transfected with the control vector, IRF1 expression construct or IRF8 expression construct. Due to the different transfection efficiencies among the expression constructs, percentages of live transfected (DAPI^-^/hrGFP^+^) cells in total live (DAPI^-^) cells were calculated in each condition, and are shown as fold changes of the calculated percentages at 6 h after transfection. ** Indicates p < 0.01 (n = 9). **E**, Overexpressed IRF1 and IRF8 were verified by immunoblotting. GFP-positive cell populations in OPC cultures transfected with the expression constructs of rat IRF1 (IRF1-GFP^+^) or IRF8 (IRF8-GFP^+^) were separated from GFP-negative cell populations (IRF1-GFP^- ^and IRF8-GFP^-^) with a cell sorter, and subjected to immunoblotting for IRF1 and IRF8, respectively. Untransfected OPCs cultured with medium alone (**C**), and medium containing IFNγ (100 ng/ml, **G**) were used as negative and positive controls, respectively.

We examined the effects of forced expression of either IRF1 or IRF8 on OPC viability. Since transient transfection of primary rat OPCs generally demonstrates limited efficiency, we used the dual expression constructs PCMV-IE-IRF1-IRES-hrGFP-pA and PCMV-IE-IRF8-IRES-hrGFP-pA in order to discriminate transfected cells from untransfected cells with the aid of coexpressed hrGFP in the transfected cells. PCMV-IE-IRES-hrGFP-pA was employed as control (Figure. 6). These dual expression constructs and the conventional cell death assay depending on the membrane-impermeable DNA-binding dye DAPI enabled us to count preapoptotic cells (TMRE^-^/DAPI^- ^cells) in either hrGFP^+ ^(transfected) or hrGFP^- ^(untransfected) population by flow cytometry with the gating strategy shown in Figure. 7B. Overexpression of IRF1 significantly increased the number of preapoptotic cells in the transfected population at 6 and 24 h after transfection. On the other hand, overexpression of IRF8 resulted in a significant increase in TMRE^-^/DAPI^-^/hrGFP^+ ^cells at 6 h, although this effect was no longer observed at 24 h. There was no significant increase in preapoptotic cells in the untransfected (hrGFP^-^) population, which could be used as an internal control, further validating these results (Figure. 7C). Moreover, total live (hrGFP^+^/DAPI^-^) OPCs overexpressing IRF1 were reduced by approximately 50% from 6 to 24 h after transfection (Figure. 7D). These results indicate that upregulation of IRF1 protein is sufficient for activation of the apoptotic pathway in OPCs, but that up-regulation of IRF8 protein alone is not.

**Figure 8:**
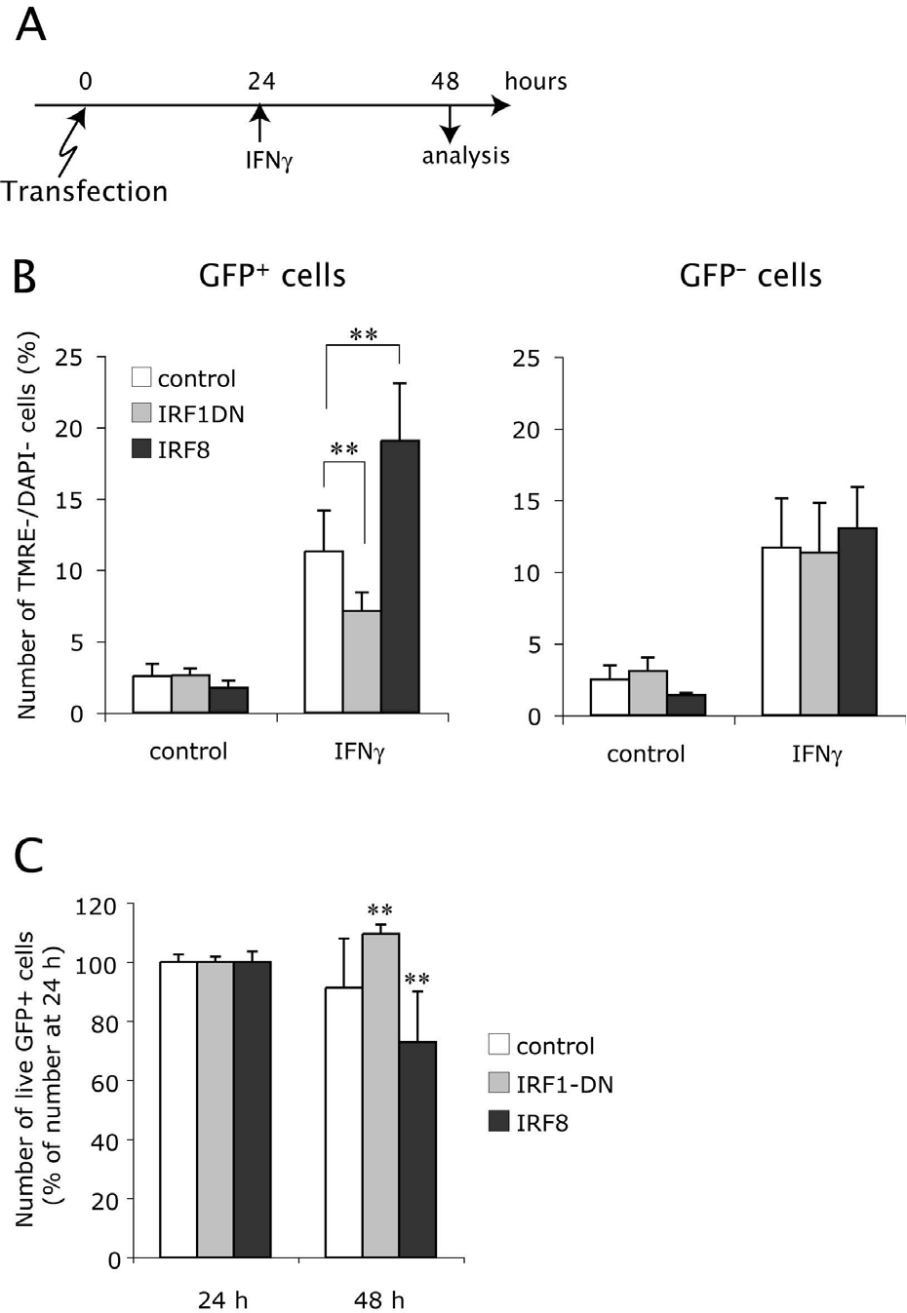
**Effects of overexpressed dominant-negative IRF1 and overexpressed IRF8 on IFNγ-induced OPC apoptosis**. **A**, OPCs were transfected by electroporation with the control vector (PCMV-IE-IRES-hrGFP-pA), the expression construct of a dominant-negative form of IRF1 (IRF1DN-hrGFP) which is a fusion protein of IRF1 DNA-binding domain (IRF1DBD) and hrGFP (PCMV-IE-IRF1DBD/hrGFP-pA), and the expression construct of rat IRF8 (PCMV-IE-IRF8-IRES-hrGFP-pA). Cells were cultured with GM for 24 h after transfection, and then treated with GM alone (control) or GM plus IFNγ (100 ng/ml). At 24 h after treatments, cells were stained with TMRE and DAPI, and analyzed by flow cytometry as in Figure. 7. **B**, Number of preapoptotic (TMRE^-^/DAPI^-^) OPCs in the cultures subjected to electroporation with the control vector or the IRF1DN expression construct at 24 h after treatment with IFNγ (100 ng/ml). Transfected (hrGFP^+^) and untransfected (hrGFP^-^) populations were analyzed separately, using the same gatings as in Figure. 7. **C**, Number of live transfected cells (DAPI^-^/hrGFP^+^) in the cultures transfected with the control vector, IRF1DN expression construct or IRF8 expression construct after a 24 h IFNγ-treatment (48 h after transfection). Percentages of DAPI^-^/hrGFP^+ ^cells in total live (DAPI^-^) cells were calculated in each condition, and are shown as fold changes of the percentages just before addition of IFNγ (24 h after transfection). Note that the same percentages of transfected (hrGFP^+^) and untransfected (hrGFP^-^) OPC populations died during 24 h after addition of IFNγ in the control group, whereas less and more transfected OPCs were dead in the IRF1-DN and IRF8 groups, respectively. ** Indicates p < 0.01 in comparison with the corresponding data at 24 h (n = 9).

To further confirm the proapoptotic effects of IRF1 on OPCs, we overexpressed a fusion protein of the IRF1 DNA-binding domain and hrGFP as a dominant negative form of IRF1 (IRF1DN-hrGFP) in OPCs. OPCs were treated with IFNγ at 24 h after transfection, and the number of preapoptotic (TMRE^-^/DAPI^-^) cells in either hrGFP^+ ^or hrGFP^- ^population was measured at 24 h after addition of IFNγ (Figure. 8A). Fluorescence microscopy demonstrated that the IRF1DN-hrGFP protein was localized in the nuclei of OPCs (Figure. 6C). Preapoptotic cells were partially but significantly reduced in the OPCs expressing IRF1DN-hrGFP at 24 h after addition of IFNγ, compared to the OPCs expressing hrGFP alone (Figure. 8B). These results confirmed that inhibition of functional IRF1 by IRF1DN-hrGFP protects OPCs from IFNγ-induced apoptosis, and that IRF1 is one of the ISGs that principally mediate IFNγ-induced OPC apoptosis.

### IRF8 enhances IFNγ-induced apoptosis of OPC

Although overexpression of IRF8 itself was not sufficient to induce OPC apoptosis, it remained to be clarified whether overexpressed IRF8 enhanced IFNγ-induced OPC apoptosis. To examine this, OPCs were transfected with PCMV-IE-IRF8-IRES-hrGFP-pA, and then treated with IFNγ at 24 h after transfection. Numbers of preapoptotic (TMRE^-^/DAPI^-^) cells were significantly increased in IRF8 overexpressing OPCs, compared with those transfected with the control vector, at 24 h after addition of IFNγ (Figure. 8B).

**Figure 9:**
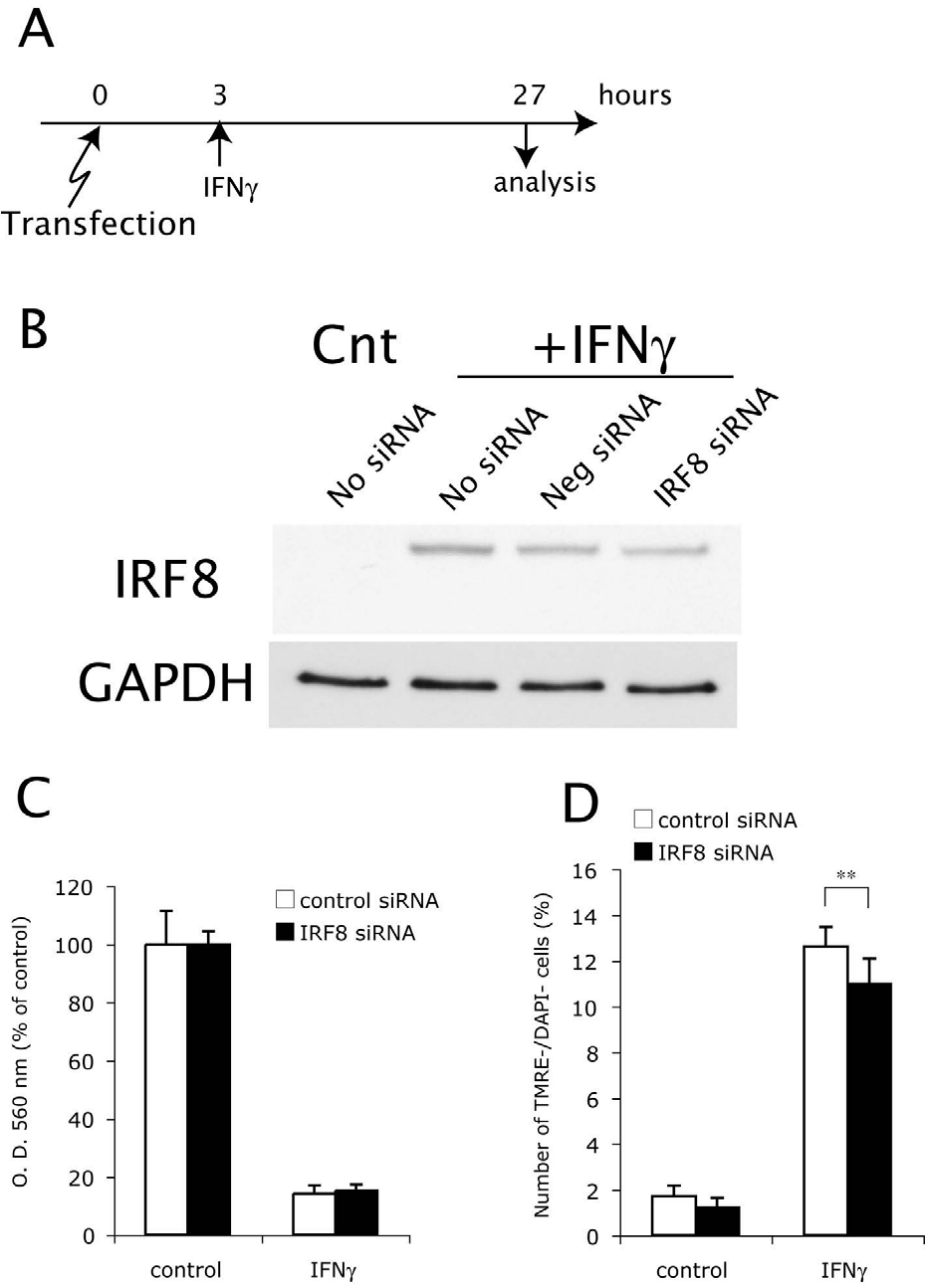
**Selective down-regulation of IRF8 by siRNA protects OPCs from IFNγ-induced OPC apoptosis**. **A**, OPCs were transfected by electroporation with IRF8 siRNA and negative conrol siRNA. Cells were cultured with GM for 3 h after transfection, and then treated with GM alone (control) or GM plus IFNγ (100 ng/ml). At 24 or 48 h after treatments, cells were subjected to western blotting. MTT assay, or stained with TMRE and DAPI followed by the flow cytometric analysis as in Figure. 7. **B**, Protein levels of IRF8 in the OPCs transfected with negative control siRNA (Neg siRNA) or IRF8 siRNA were examined by immunoblotting at 24 h after addition of IFNγ (+IFNγ, 100 ng/ml). The untransfected OPCs (No siRNA) treated with IFNγ (+IFNγ, 100 ng/ml) or medium alone (C) for 24 h were used as positive and negative control for IRF1 expression, respectively. B, Viability of the OPCs transfected with negative control siRNA (control siRNA, open bar) or IRF8 siRNA (closed bar) was measured by MTT assay at 48 h after treatment with GM alone (control) or GM plus IFNγ (100 ng/ml). **C**, Number of preapoptotic (TMRE^-^/DAPI^-^) OPCs in the cultures subjected to electroporation with negative control siRNA (control siRNA, open bar) or IRF8 siRNA (closed bar) at 24 h after treatment with IFNγ (100 ng/ml). ** Indicates p < 0.01 (n = 9).

We further tested whether down-regulation of IRF8 by siRNA protected OPCs from IFNγ-induced apoptosis. Immunoblots after introduction of siRNA for IRF8 demonstrated that the employed siRNA only partially inhibited IRF8 induction by IFNγ (Figure. 9B). The OPCs with reduced IRF8 protein levels to this extent showed no significant improvement in the viability of OPCs compared with those transfected with control siRNA at 48 h after treatment with IFNγ (Figure. 9C). Nevertheless, the number of TMRE^-^/DAPI^- ^preapoptotic OPCs was partially but significantly decreased in the cultures transfected with IRF8 siRNA than that in the control cultures at 24 h after treatment with IFNγ (Figure. 9D).

**Figure 10:**
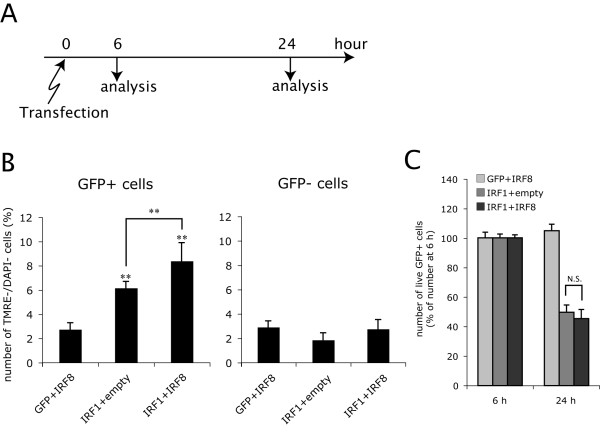
**IRF8 enhances the proapoptotic effect of IRF1 in the absence of IFNγ**. **A**, OPCs were co-transfected with the following combinations of expression constructs by electroporation; The control GFP vector (PCMV-IE-IRES-hrGFP-pA) plus the rat IRF8 expression construct without GFP reporter (PCMV-IE-IRF8-pA) (GFP+IRF8), the rat IRF1 expression construct with GFP reporter (PCMV-IE-IRF1-IRES-hrGFP-pA) plus the rat IRF8 expression construct without GFP reporter (PCMV-IE-IRF8-pA) (IRF1+IRF8), or PCMV-IE-IRF1-IRES-hrGFP-pA plus the empty vector (PCMV-IE-pA) (IRF1+empty). These OPCs were incubated with TMRE and DAPI at 6 and 24 h after transfection and analyzed by flow cytometry. **B**, Number of preapoptotic (TMRE^-^/DAPI^-^) OPCs at 24 h after electroporation with GFP+IRF8, IRF1+empty, and IRF1+IRF8. Transfected (hrGFP^+^) and untransfected (hrGFP^-^) populations were analyzed separately, using the same gatings as in Figure. 7. ** Indicates p < 0.01 compared with GFP^- ^(non-transfected) counterparts, or in comparison between the two groups indicated. **C**, Reduction in live transfected cells (DAPI^-^/hrGFP^+^) at 24 h in the cultures transfected with GFP+IRF8, IRF1+empty, or IRF1+IRF8. Percentages of DAPI^-^/hrGFP^+ ^cells in total live (DAPI^-^) cells were calculated in each condition, and are shown as fold changes of those at 6 h after transfection. N.S. indicates p > = 0.05 between the two groups indicated.

Furthermore, we examined whether IRF8 enhances the proapoptotic effects of overexpressed IRF1 in OPCs in the absence of IFNγ. OPCs were co-transfected with the IRF1 expression construct with hrGFP reporter (PCMV-IE-IRF1-IRES-hrGFP-pA) and an IRF8 expression construct without hrGFP reporter (PCMV-IE-IRF8-pA) by electroporation to facilitate identification of double transfected cells (Figure. 10). As far as we could determine by immunocytochemistry, 96 ± 4% (n = 3) of hrGFP^+ ^cells were positive for IRF8 immunoreactivity, and 85 ± 5% (n = 3) of IRF8^+ ^cells were hrGFP^+ ^at 6 h after transfection, confirming that virtually all hrGFP^+ ^cells expressed both IRF1 and IRF8 after co-transfection. When both IRF8 and IRF1 were overexpressed in OPCs (IRF1+IRF8), preapoptotic (TMRE^-^/DAPI^-^) cells were significantly more than those in the OPCs overexpressing IRF1 alone (IRF1+empty) (Figure. 10B), although there was no statistical significance in reduction of live transfected cells (hrGFP^+^) between the IRF1+empty and IRF1+IRF8 groups at 24 h after transfection (Figure. 10C). These results indicated that overexpressed IRF8 protein directly enhances the proapoptotic effects of IRF1 in OPCs even in the absence of IFNγ.

## Discussion

Proapoptotic effects of IFNγ and at most minimal cytotoxic effects of IFNβ on OPCs have been reported previously [4-9,12,13]. In the present study, however, we have directly compared effects of IFNγ and IFNβ on OPCs in the same *in vitro *condition, and confirmed a substantial difference in proapoptotic effects between the two IFNs. Furthermore, IFNβ was not protective against IFNγ-induced OPC apoptosis, despite several prior reports that IFNβ antagonizes IFNγ signaling [39-43]. As far as we could determine by transcriptional induction of IRF1, simultaneous application of IFNβ failed to reduce IFNγ-mediated robust induction of IRF1 [17]. Although the mechanisms underlying the beneficial therapeutic effects of IFNβ on relapsing-remitting MS are still largely unknown, recent studies have indicated that IFNβ and type I IFN receptor-mediated signaling limit CNS autoimmunity by regulating innate immune responses in peripheral tissues [44,45] and the production and properties of T_H_17 cells, a pathogenic T helper subset largely responsible for CNS autoimmunity [46]. Despite the beneficial effects of IFNβ which is further ensured by far less cytotoxicity of IFNβ to OPCs, we observed that IFNβ did inhibit the cell cycle in OPCs, though to a lesser extent than IFNγ. It is thus conceivable that, as demonstrated by Trebst et al.[14], IFNβ attenuates the endogenous capability for remyelination, which is presumably masked by its profound beneficial effects on the immune system.

Based on the marked difference in proapoptotic effects between IFNγ and IFNβ on OPCs, our next aim in this study was to identify those ISGs responsible for IFNγ-mediated OPC apoptosis. IFNγ induces robust and sustained elevation of IRF1, whereas IFNβ elicits only a transient elevation of IRF1, which ends up being undetectable at the protein level at 24 h after treatment, indicating that IRF1 is a candidate for such an ISG [17]. In support of this, Balabanov's group has recently reported that, using a lentiviral expression system, down-regulation of IRF1 by IRF1 shRNA partially protected against IFNγ-induced OPC apoptosis, and that forced expression of IRF1 reduced the viability of OPCs [18]. We employed a different forced expression system and a dominant negative approach in this study, and confirmed significant involvement of IRF1 in IFNγ-mediated OPC apoptosis. We further provided direct evidence for activation of the mitochondrial apoptotic pathway by overexpression of IRF1 alone. Notably, however, both approaches used in our study and the study by Balabanov's group to down-regulate IRF1-mediated transcription failed to completely inhibit IFNγ-mediated apoptotic events, suggesting possible functional redundancy of the ISGs involved in IFNγ-mediated transcriptional activation leading to apoptosis of OPCs. Given the structural and functional similarity among members of the IRF family and their known interactions, transcriptional activity of IRF1 is likely to be modified or compensated by the other members of the IRF protein family.

In an effort to obtain a comprehensive expression profile of the IRF family in OPCs stimulated by either IFNγ or IFNβ, we found that IRF8 was also up-regulated by IFNγ but not by IFNβ. IRF8 was originally identified as a protein that binds to the ISRE in the promoter region of the MHC class I gene H-2LD [47], and was believed to be expressed exclusively in the hematopoietic lineage (Reviewed in [48]). Our result indicates that OPCs are also capable of expressing IRF8 in response to IFNγ. In contrast to overexpression of IRF1, however, overexpression of IRF8 alone resulted in only transient depolarization of the mitochondrial membrane in OPCs, but failed to reduce their viability. More importantly, despite this weak proapoptotic effect of overexpressed IRF8 itself, it significantly enhanced the IFNγ-induced apoptosis and proapoptotic effect of overexpressed IRF1 in OPCs even in the absence of IFNγ. Unlike other IRF members, IRF8 is capable of binding to the target DNA motif only following association with IRF1, IRF2 or non-IRF transcription factors such as PU.1 [36,49]. As an example, IRF8 and IRF1 synergistically induce several genes, such as IL-12 and iNOS [50,51], in activated macrophages. A study from Ozato's group also demonstrated that IRF8 induced by activated STAT1 forms a multiprotein transcriptional complex with other nuclear proteins, which binds to GAS, and, in turn, potentiates transcriptional activation of the ISGs in a GAS-dependent manner [52]. Therefore, it is conceivable that, although IRF8 alone is not sufficient to activate the apoptogenic cascade in OPCs, IRF8 enhances IFNγ-induced OPC apoptosis by interacting with other transcription factors activated by IFNγ. Indeed, IRF8 is known to function as a proapoptotic transcription factor like IRF1. IRF8-deficient mice are characterized by a myeloproliferative phenotype resulting in a syndrome similar to human chronic myelogenous leukemia [53]. This oncogenic phenotype is attributable to cytokine hypersensitivity and apoptosis resistance of IRF8 deficient myeloid progenitor cells [54]. During differentiation of the myeloid lineage, IRF8 down regulates anti-apoptotic genes such as Bcl-X_L_, one of anti-apoptotic member of the Bcl-2 family, and PTPN13, which encodes an inhibitor of Fas-mediated apoptosis [55,56]. The anti-oncogenic roles of IRF8 are associated with its proapoptotic function in the other types of tumors as well. In colon carcinoma cells, IRF8 induced by IFNγ sensitizes them to Fas-mediated apoptosis, but the silencing of the IRF8 gene by methylation of its promoter region renders them resistance to IFNγ-mediated apoptosis [57].

Reduction of IRF8 by siRNA failed to enhance viability of OPCs after treatment with IFNγ, although it partially but significantly decreased the number of preapoptotic cells. However, we still could not rule out a contribution of the endogenous IRF8 in the IFNγ-induced OPC apoptosis, because the transfection of the IRF8 siRNA resulted in only a partial suppression against the robust IRF8 induction by IFNγ. Together, these results support the notion that endogenous IRF8 positively regulates the IFNγ-induced OPC apoptosis depending on its induced dosage.

We previously demonstrated that, unlike OPCs, mature myelin-producing oligodendrocytes were totally resistant to IFNγ-induced apoptosis [10]. Nevertheless, IRF1 was similarly induced by IFNγ in mature oligodendrocytes compared with OPCs [10,17]. We also confirmed that IFNγ induced IRF8 mRNA at similar levels in both OPCs and mature oligodendrocytes (Data not shown.). These results indicate that IRF1-mediated transcriptional activations may be necessary to activate the apoptotic cascade in OPCs, but are not sufficient. We speculate that differences in cellular context between OPCs and mature oligodendrocytes such as activities of ERK signaling are the other necessary components for IFNγ-induced OPC apoptosis as well [10,58,59].

## Conclusions

Conclusions from this study are summarized as follows. First, unlike IFNγ, IFNβ is far less capable of inducing OPC apoptosis. Second, our comprehensive analysis of the IRF family members in IFNγ- and IFNβ-treated OPCs identified that IRF1 and IRF8 are preferentially up-regulated by IFNγ. Third, functional analyses of IRF1 and IRF8 revealed that not only IRF1 but also IRF8 contribute to the IFNγ-mediated OPC apoptosis. This finding will help us to identify downstream genes involved in OPC apoptosis. These transcription factors and their downstream target genes could be potential therapeutic targets to enhance remyelination in MS.

## Competing interests

The authors declare that they have no competing interests.

## Authors' contributions

MH prepared the cultures and carried out most of the experiments and data analysis, and wrote the manuscript. AI carried out RNA isolation and qPCR experiments, and helped development of the expression constructs. DP participated in data interpretation and critical reading of the manuscript. KO provided anti-IRF8 antibody and participated in data interpretation and critical reading of the manuscript. TI conceived the study, contributed to the experimental design, and helped to write the manuscript. All authors read and approved the final manuscript.
